# Supplementary material for: Human-centered design of a health recommender system for orthopaedic shoulder treatment
Source: BMC Med Inform Decis Mak. 2025 Jan 10;25:17. doi: 10.1186/s12911-025-02850-x (PMC11720343; doi:10.1186/s12911-025-02850-x)
Supplement: Supplementary file 1 — Supplementary Material 1 [file 12911_2025_2850_MOESM1_ESM.docx]

**INTERVIEW GUIDE**

Participant ID:

Time of interview:

Date:

Interviewer:

Good morning/afternoon,

Thank you for agreeing to take part in this interview.

- My name is Akanksha Singh and I am a graduate student at USC.
- My name is Sarah Floyd and I am an Assistant Professor at Clemson University.

The goal of this interview is to get your feedback on a prototype of a new Health Recommender System (HRS) concept in orthopaedic medicine. Recommender Systems (RSs) are software tools and techniques that provide suggestions for items of interest to a user. For a Health Recommender System, the item of interest is a piece of non-confidential medical information. The prototype HR system has been developed for the use case of proximal humerus fracture. Currently this system only exists in prototype form and is early in conceptualization. We believe that with valuable feedback from physicians like yourself, we could work to refine and further develop a useful system that could deliver personalized data to help you make evidence-based treatment decisions for your patients.

We’d like to begin the interview now, is it OK if we begin recording now? [If yes, turn on record in Zoom]

I’d like to start by asking you a few questions about yourself. These answers are for our research purposes and will not be shared outside the research team.

What is your age group:

- 25-34
- 35-44
- 45-54
- 55-64
- 65+

Gender: How do you identify?

- Man
- Woman
- Non-binary
- Prefer to self-describe (open response)

How many years have you been in practice?

- Under 5
- 5-10 years
- 10-15 years
- 15-20 years
- 20-25 years
- 25-30 years
- 30+ years

There will be three parts to this interview. The first part will focus on treatment decision-making for PHF and other similar orthopaedic conditions. The second part of the interview will be where we present a use case example in the health recommender system and discuss your reactions. The third and final part will be a series of questions about your experience. The interview will total approximately 45-55 minutes.

Now, please respond in any way you like to any question. There are no right or wrong answers, whatever is right for you is the correct answer. You may decline to answer any questions. And you may discontinue the interview at any time. When you respond, keep talking until you have shared all the thoughts and feelings that you have.

**PART ONE**

Ok, we are now going to ask a few questions about how you currently make treatment decisions for patients with proximal humerus fractures (PHF).

**PHF Treatment Decision-making**

1. Please begin by walking us through your patient evaluation and decision-making processes when first seeing a patient with PHF?
2. What treatment options do you consider for PHF patients and what time frames for those options are relevant?
3. Do you ever discuss your treatment decision making with your physician peers?
4. Do you ever review or evaluate your historical treatment decision making patterns?
5. How would you define treatment success for a patient with PHF?
6. What Patient Reported Outcomes (PROs) would you want to/do you collect?
7. Do you currently use any clinical decision support or health recommender systems to assist in treatment decision making?

**PART TWO**

Ok, we will now focus on examples of use cases in the HRS and ask you to respond to questions about usability

I will start by providing a description of the layout of the system and then proceed to the questions.

We have named this HRS, ICIT which stands for *Informatics Consult for Individualized Treatment*. The goal of ICIT is to allow physicians to query historical EHR records for similar patients to the one they are currently treating. Historical records in the EHR could be queried and summarized to show treatment rates and outcomes for patients who chose different treatment options. The top right-hand corner shows the physician user. A user profile would exist for each physician in the practice. On the left land side is the patient search criteria where tailored patient searches can occur. The middle panes and tables display treatment rates and outcomes.

**Initial reaction:**

1. Imagine this interface was embedded in the EHR and readily available within the clinical encounter/workflow. What are your first impressions of the HRS visual design?

**Patient Search Criteria:**

**I am going to apply search criteria for a simulated patient. Let’s begin by selecting Dr. Schooleys’s user profile. Ok, let’s pretend you are Dr. Schooley and he is treating a 75-year old female patient. You might search for patients 70-80 to provide a range. You’ll see the search filters apply on the top of the middle pane to display the criteria applied. We will say she has a displaced fracture.**

**By not enabling the toggle on a filter, all patients are included in the search, who may or may not have that condition.**

**The reason we have added these patient selection criteria, is that we are trying to identify a similar group of historical patients that match the target patient profile.**

1. Which of these patient characteristics are most useful in context of PHF treatment decisions, or if there is something missing, what would you want to add?
2. With AI advances it would be possible to automate the cohort matching process, would you prefer to automate the display of the results or have the ability to toggle filters to tailor your search or some combination of both?

**Treatment Comparators:**

**Ok, we are going to look at the middle pane now. At the top are two dials that display the treatment rates for this cohort of patients. So, for these XXX patients, xxx% were treated conservatively and yyy% received initial surgery. The middle figure shows the trend in initial surgery rate over time and has a line for Dr. Floyd’s practice patterns as well as the average of all other surgeons in the practice. The bottom two pie charts display Dr. Floyd’s surgery rate compared to High-volume surgeons in the practice. High volume surgeons are defined as those physicians in the practice who treated the largest proportion of the matched sample.**

1. Is it helpful to see the treatment rate and trend in treatment rate over time?
2. Would it be helpful to see how you have treated similar patients in the past?
3. Would it be helpful to see how high-volume physicians treat similar patients?
4. Do you have any suggestions about additional visuals would you like to see?

**Patient-Reported Outcomes:**

**This tab shows the PRO results for each treatment group within the matched cohort. We display SANE and Constant scores, PROMIS Physical Function, Pain and Forward flexion at 3, 6, 12 and 24 months. The purple bars and numbers are the average scores at each outcome period for the initial surgery group and the blue are the scores for the conservative management group.**

1. Which patient-reported outcome results are most helpful to see?
2. Do you feel like the patient-reported outcome results could be helpful in conversations with expected outcomes for patients?
3. Do you have any recommendations on how to improve the layout?
4. Do you have any suggestions about additional visuals would you like to see?

**Healthcare Utilization Outcomes Tab:**

**This tab shows healthcare utilization for each treatment group within the matched cohort. In the upper left hand corner, .. [Explain the various numbers and visuals on the page] ..**

**.. utilization period reflects one year period after the treatment.**

1. Do you think this type of information would be valuable to patients when setting expectations for different treatment options?
2. Are there any additional visuals you would like to see here?

**General Usability and Necessity:**

1. [Clinical] This specific system is designed for PHF. What PHF patients do you see this system being valuable for?
   1. For about what percentage of patients with PHF is there NOT an obvious treatment choice?
      1. What characteristics describe this patient?
2. How would you describe this system to a colleague?
3. Please describe your general feelings about using a system like this.
4. What barriers would prevent you from using the HRS system?
5. During a patient visit, when do you see yourself using this HRS system?
6. How long would you have to look at a system like this in practice?
7. Who do you think would benefit most from the use of the HRS system? (i.e., low-volume providers, newer providers, patients, etc.)
   1. How might this system facilitate shared decision-making with the patient?
8. Do you have any final suggestions for improving the HRS?

**PART THREE**

We have now completed the use case and would like to conclude with asking you to complete a survey. Please use the link we just provided in the email to complete the short survey. Please use your Participant ID provided in your email. We will stay connected here in case you have any questions, and please let us know when you are finished.

[Participate will respond to these survey questions independently]

In thinking about the HRS you just previewed, please say whether you agree or disagree with the following statements:

| Custom Measures for ICSCEDIT | | | | | | | | | |
| --- | --- | --- | --- | --- | --- | --- | --- | --- | --- |
|  | Strongly Agree Strongly Disagree | | | | | | | | |
| Q1. This system was easy to navigate. | 5 | | 4 | | 3 | | 2 | | 1 |
| Q2. This system was easy to understand. | 5 | | 4 | | 3 | | 2 | | 1 |
| Q3. I found this system useful in making informed treatment decisions. | 5 | | 4 | | 3 | | 2 | | 1 |
| Q4. The healthcare data was easy to interpret. | 5 | | 4 | | 3 | | 2 | | 1 |
| Q5. The patient cohort search measures were easy to understand. | 5 | | 4 | | 3 | | 2 | | 1 |
| Q6. Given the directions provided on the system, I felt like I would know what to do. | 5 | | 4 | | 3 | | 2 | | 1 |
| Q7. The system would help me in making data informed treatment decisions with my patients with Proximal Humerus Fractures. | 5 | | 4 | | 3 | | 2 | | 1 |
| Q8. I would be willing to use this system to discuss treatment options and possible treatment outcomes with my Proximal Humerus Fracture patients. | 5 | | 4 | | 3 | | 2 | | 1 |
| Q9. This data displayed included the important factors to consider when selecting treatment options for Proximal Humerus Fractures patients. | 5 | | 4 | | 3 | | 2 | | 1 |
| Q10. I would be willing to use a similar system integrated in EHR in my day-to-day work for other orthopaedic conditions. | 5 | | 4 | | 3 | | 2 | | 1 |
| In thinking about the technology that you used, please respond to the following: (SUS) | | | | | | | | | |
|  | | Strongly Agree Strongly Disagree | | | | | | | |
| I would like to use this system frequently. | | 5 | | 4 | | 3 | | 2 | 1 |
| I found this system unnecessarily complex. | | 5 | | 4 | | 3 | | 2 | 1 |
| I thought this system was easy to use. | | 5 | | 4 | | 3 | | 2 | 1 |
| I thought that I would need assistance to be able to use this system. | | 5 | | 4 | | 3 | | 2 | 1 |
| I found the various functions in this system were well integrated. | | 5 | | 4 | | 3 | | 2 | 1 |
| I thought there was too much inconsistency in this system. | | 5 | | 4 | | 3 | | 2 | 1 |
| I would imagine that most people would learn to use this system very quickly. | | 5 | | 4 | | 3 | | 2 | 1 |
| I found this system very cumbersome/awkward to use. | | 5 | | 4 | | 3 | | 2 | 1 |
| I felt very confident in using this system. | | 5 | | 4 | | 3 | | 2 | 1 |
| I needed to learn a lot of things before I could get going with this system. | | 5 | | 4 | | 3 | | 2 | 1 |

Thank you so much for your time.
